# Supplementary material for: Transcriptome Profiling in the Hippocampi of Mice with Experimental Autoimmune Encephalomyelitis
Source: Int J Mol Sci. 2022 Nov 27;23(23):14829. doi: 10.3390/ijms232314829 (PMC9738199; doi:10.3390/ijms232314829)
Supplement: Supplementary file 1 [file ijms-23-14829-s001.zip › Suppl_Figures_M-W et al.pdf]

International Journal of
Molecular Sciences

MDPI

Supplementary Figures

Transcriptome Profiling in the Hippocampi of Mice with Experimental Autoimmune Encephalomyelitis

Poornima D. E. Weerasinghe-Mudiyanselage [1,†], Sohi Kang [1,†], Joong-Sun Kim [1], Sung-Ho Kim [1], Hongbing Wang [2], Taekyun Shin [3], Changjong Moon [1,*]

[1] Department of Veterinary Anatomy and Animal Behavior, College of Veterinary Medicine and BK21 FOUR Program, Chonnam National University, Gwangju 61186, Korea
[2] Department of Physiology and Neuroscience Program, Michigan State University, MI 48824, USA
[3] Department of Veterinary Anatomy, College of Veterinary Medicine and Veterinary Medical Research Institute, Jeju National University, Jeju 63243, South Korea
* Correspondence: moonc@chonnam.ac.kr; Tel: +82-62-530-2838
† First two authors (P.D.E.W-M. and S.K.) contributed equally to this work.

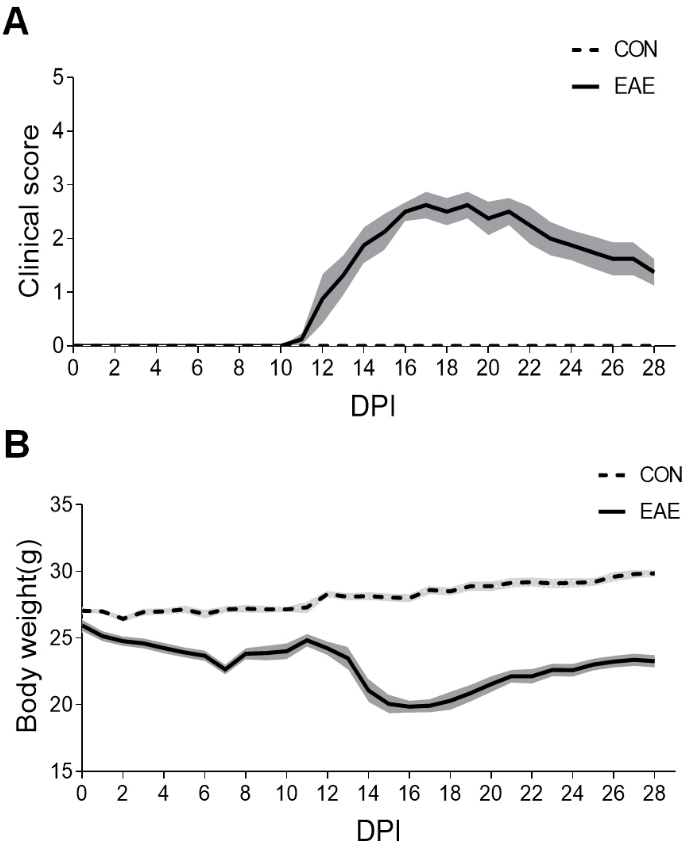

Figure S1. Clinical score (A) and body weight (B) of EAE-affected mice. Clinical symptoms were scored according to a 0–5 scale. Data are presented as the mean values of paralysis scores ± SEM (n = 10/group). CON, control group; DPI, day post-immunization; EAE, EAE-affected group.

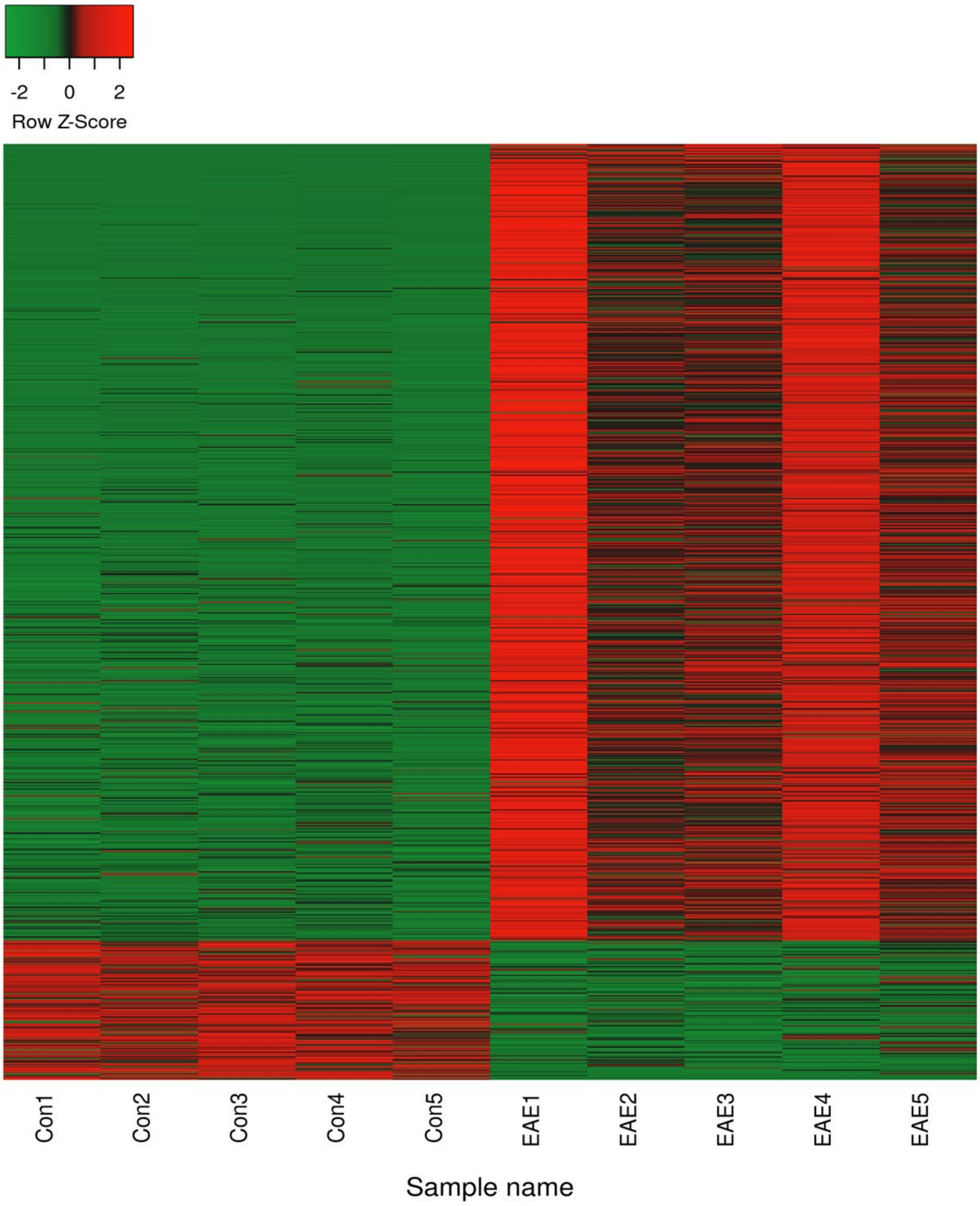

Figure S2. Heat map of differentially expressed genes (DEGs) in the hippocampi of CON vs. EAE-affected mice. Green and red indicate low and high expressions, respectively. Expression data are represented as Z-score for 1202 DEGs. CON, control group; EAE, EAE-affected group.

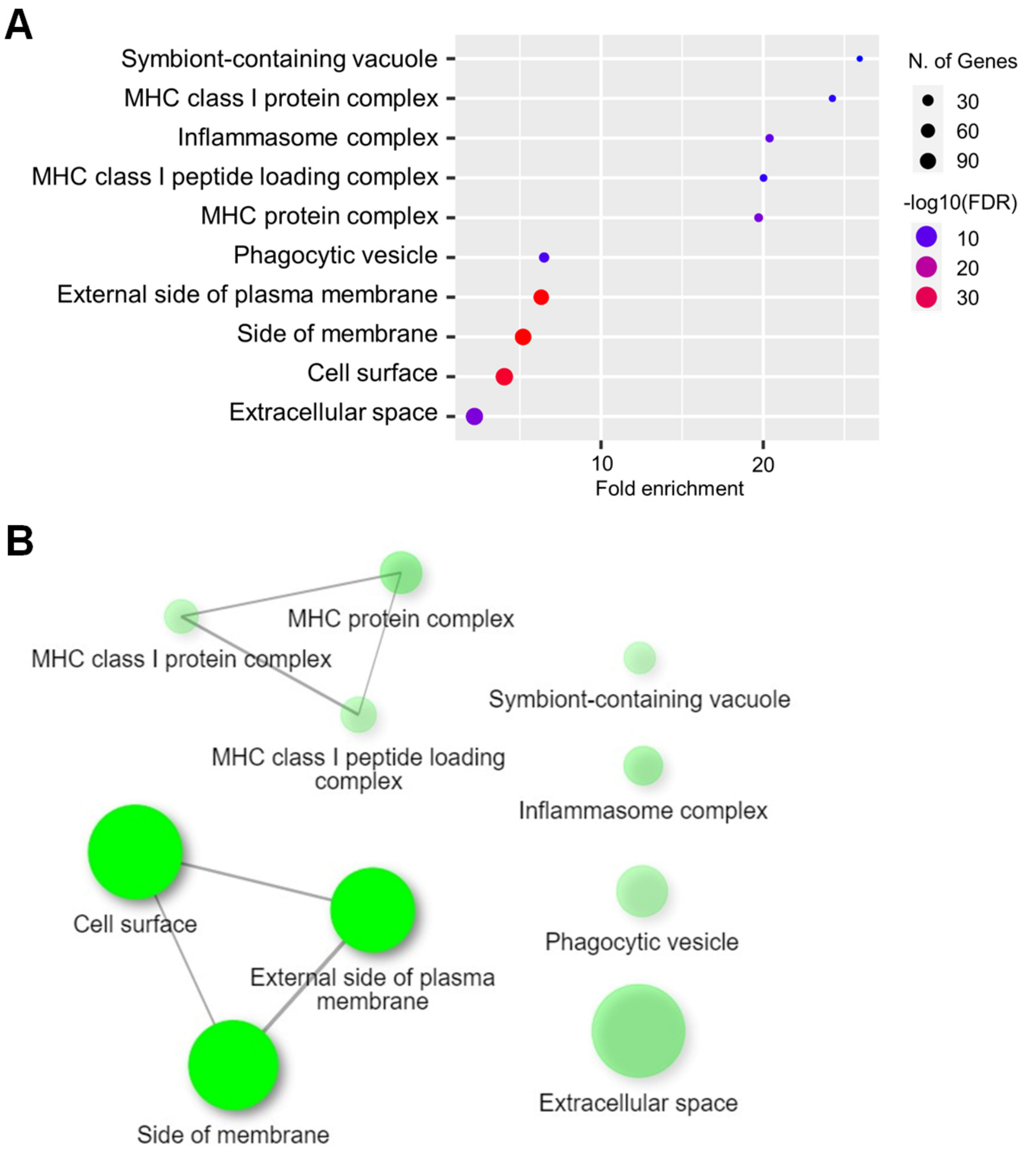

Figure S3. (A) Dot plot of enriched genes under GOCC (top ten) in EAE-affected hippocampi with > 1.5-fold change and *p*-value < 0.05. (B) Interactive plot showing the relationship between enriched pathways.

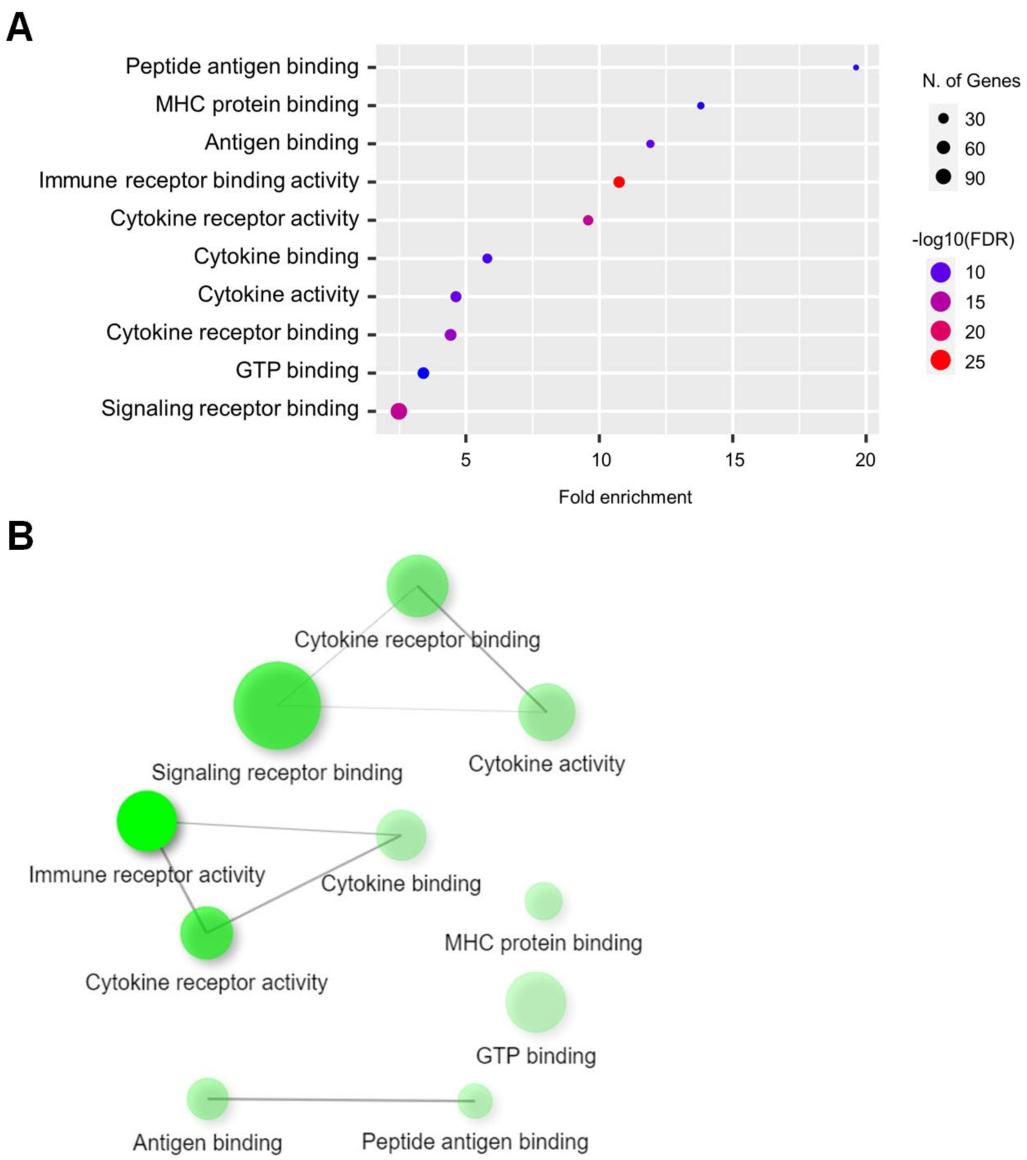

Figure S4. (A) Dot plot of enriched genes under GOMF (top ten) in EAE-affected hippocampi with > 1.5-fold change and p-value < 0.05. (B) Interactive plot showing the relationship between enriched pathways.

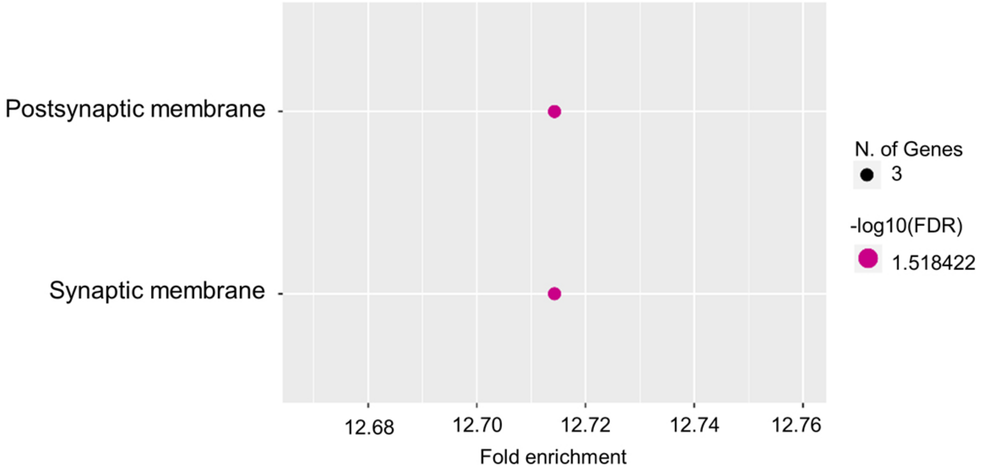

Figure S5. Dot plot of enriched genes under GOCC in EAE-affected hippocampi with > −1.5-fold change and p-value < 0.05.

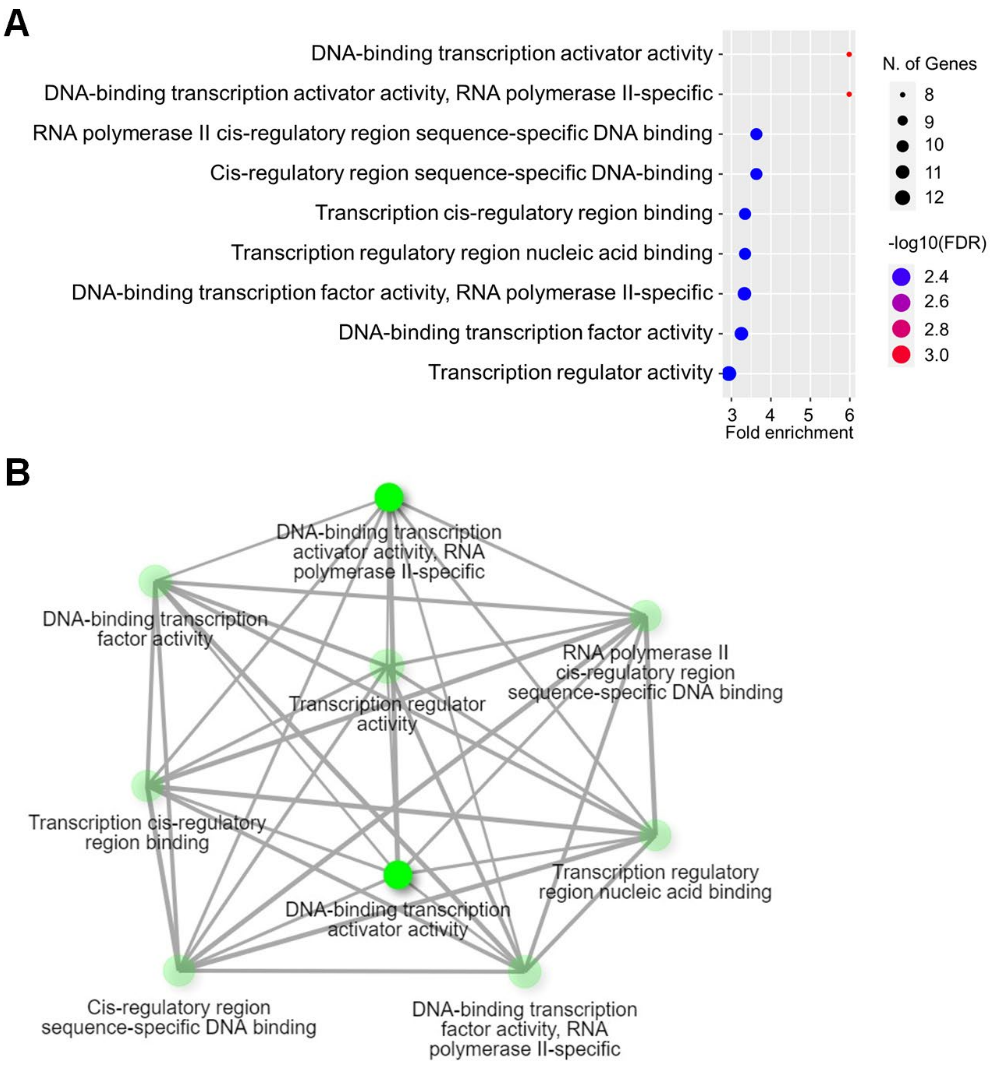

Figure S6. (A) Dot plot of enriched genes under GOMF in EAE-affected hippocampi with > −1.5-fold change and p-value < 0.05. (B) Interactive plot showing the relationship between enriched pathways.

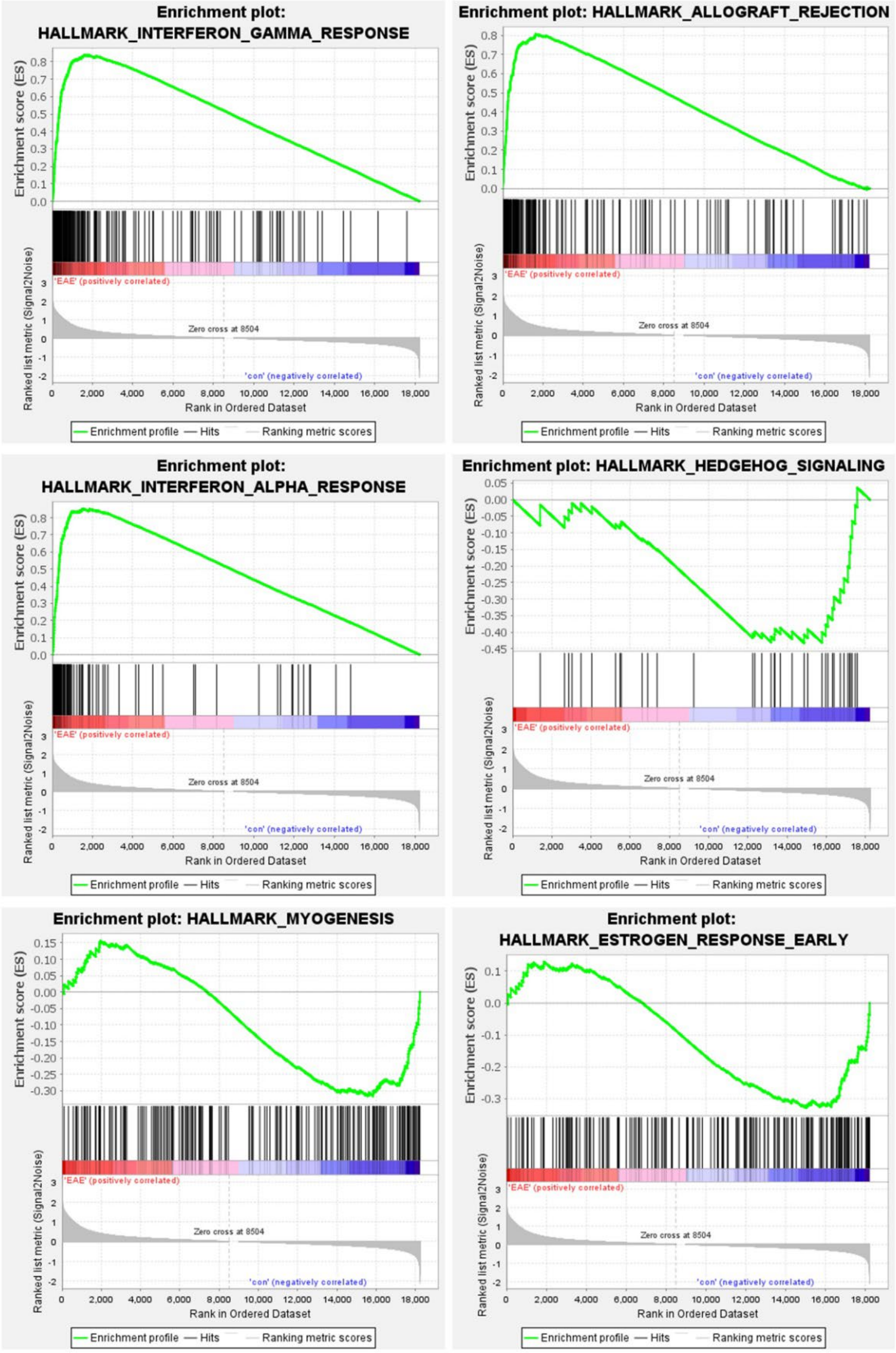

Figure S7. Representative enrichment plots for positively and negatively enriched hallmark gene sets in the hippocampi of mice with EAE.

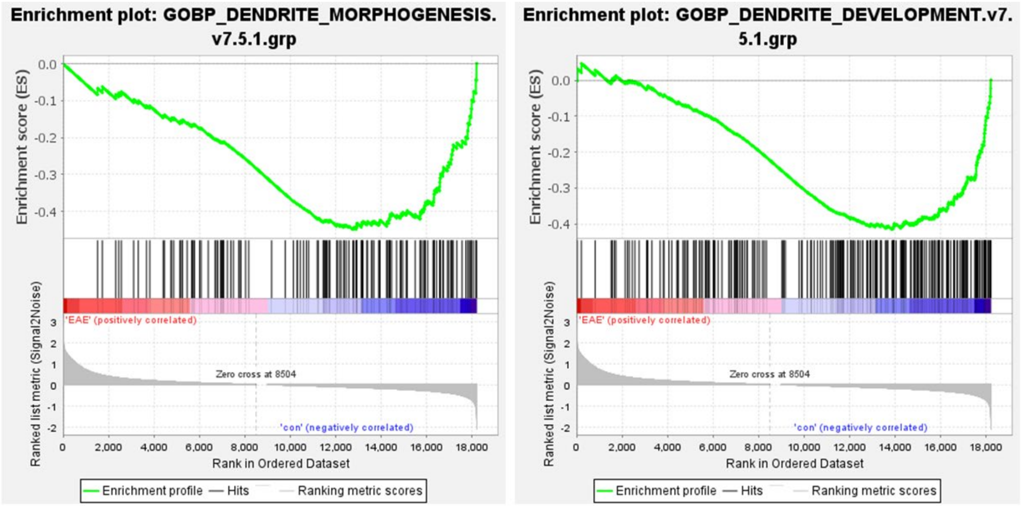

Figure S8. Representative enrichment plots for neuroplasticity-related curated gene sets in the hippocampi of mice with EAE.
