# Supplementary material for: Transcriptome Profiling in the Hippocampi of Mice with Experimental Autoimmune Encephalomyelitis
Source: Int J Mol Sci. 2022 Nov 27;23(23):14829. doi: 10.3390/ijms232314829 (PMC9738199; doi:10.3390/ijms232314829)
Supplement: Supplementary file 1 [file ijms-23-14829-s001.zip › Suppl_Tables_M-W et al.pdf]

International Journal of *Molecular Sciences*

MDPI

Supplementary Tables

Transcriptome Profiling in the Hippocampi of Mice with Experimental Autoimmune Encephalomyelitis

Poornima D. E. Weerasinghe-Mudiyanselage [1,†], Sohi Kang [1,†], Joong-Sun Kim [1], Sung-Ho Kim [1], Hongbing Wang [2], Taekyun Shin [3], Changjong Moon [1,*]

[1] Department of Veterinary Anatomy and Animal Behavior, College of Veterinary Medicine and BK21 FOUR Program, Chonnam National University, Gwangju 61186, Korea

[2] Department of Physiology and Neuroscience Program, Michigan State University, MI 48824, USA

[3] Department of Veterinary Anatomy, College of Veterinary Medicine and Veterinary Medical Research Institute, Jeju National University, Jeju 63243, South Korea

* Correspondence: moonc@chonnam.ac.kr; Tel: +82-62-530-2838

† First two authors (P.D.E.W-M. and S.K.) contributed equally to this work.

Table S1. Gene ontology analysis of upregulated genes in the hippocampi of EAE-induced mice under GOBP, GOCC, and GOMF.

Pathway	Fold Enrichment	Enrichment FDR	nGenes	Pathway Genes
**GOBP**				
Regulation of immune response	1.078943571	0.001373061	168	169
Regulation of response to external stimulus	1.077722432	0.012098849	141	142
Leukocyte activation	1.072048481	0.013115531	161	163
Innate immune response	1.071966275	0.013115531	160	162
Regulation of immune system process	1.071510119	0.000269318	232	235
Immune response	1.070797184	3.07E-06	294	298
Defense response to other organism	1.069085366	0.004495074	197	200
Defense response	1.06504065	0.000386969	262	267
Positive regulation of response to stimulus	1.064290789	0.012098849	202	206
Immune system process	1.061577013	3.07E-06	357	365
**GOCC**				
Symbiont-containing vacuole	25.947663	2.08E-08	7	9
MHC class I protein complex	24.262749	4.58E-09	8	11
Inflammasome complex	20.387449	3.07E-11	11	18
MHC class I peptide loading complex	20.016768	4.27E-09	9	15
MHC protein complex	19.713484	4.72E-13	13	22
Phagocytic vesicle	6.4869157	4.96E-10	21	108
External side of plasma membrane	6.309462	1.49E-38	80	423
Side of membrane	5.1907341	8.03E-39	96	617
Cell surface	4.0350577	1.62E-35	112	926
Extracellular space	2.1932326	4.34E-13	109	1658
**GOMF**				
Peptide antigen binding	19.624283	9.20E-10	10	17
MHC protein binding	13.804668	1.44E-09	12	29
Antigen binding	11.914743	7.86E-11	15	42
Immune receptor activity	10.743463	5.06E-26	38	118
Cytokine receptor activity	9.5824955	5.10E-17	27	94
Cytokine binding	5.8019618	3.88E-10	24	138
Cytokine activity	4.6257238	3.86E-11	33	238
Cytokine receptor binding	4.4265777	2.70E-13	41	309
GTP binding	3.4059946	2.22E-09	39	382
Signaling receptor binding	2.4876587	5.10E-17	112	1502

Abbreviations: GOBP, gene ontology biological process; GOCC, gene ontology cellular component; GOMF, gene ontology molecular function

Table S2. Gene ontology analysis of downregulated genes in the hippocampi of EAE-induced mice under GOBP, GOCC, and GOMF.

Pathway	Fold Enrichment	Enrichment FDR	nGenes	Pathway Genes
**GOBP**				
Learning	12.71428571	0.009452057	4	4
Skeletal muscle cell differentiation	12.71428571	0.009452057	4	4
Olefinic compound metabolic proc.	12.71428571	0.0427895	3	3
Cellular hormone metabolic proc.	12.71428571	0.0427895	3	3
Embryonic skeletal system development	12.71428571	0.0427895	3	3
Skeletal muscle tissue development	8.476190476	0.0427895	4	6
Learning or memory	7.063492063	0.0427895	5	9
Behavior	5.448979592	0.0427895	6	14
Nervous system proc.	3.632653061	0.009452057	12	42
System proc.	3.036247335	0.009452057	16	67
**GOCC**				
Postsynaptic membrane	12.71428571	0.030309434	3	3
Synaptic membrane	12.71428571	0.030309434	3	3
**GOMF**				
DNA-binding transcription activator activity	5.983193277	0.000842435	8	17
DNA-binding transcription activator activity, RNA polymerase II-specific	5.983193277	0.000842435	8	17
RNA polymerase II cis-regulatory region sequence-specific DNA binding	3.632653061	0.004183115	10	35
Cis-regulatory region sequence-specific DNA binding	3.632653061	0.004183115	10	35
Transcription cis-regulatory region binding	3.345864662	0.004200387	10	38
Transcription regulatory region nucleic acid binding	3.345864662	0.004200387	10	38
DNA-binding transcription factor activity, RNA polymerase II-specific	3.329931973	0.004183115	11	42
DNA-binding transcription factor activity	3.252491694	0.004183115	11	43
Transcription regulator activity	3.4059946	2.22E-09	39	382
Signaling receptor binding	2.4876587	5.10E-17	112	1502

Abbreviations: GOBP, gene ontology biological process; GOCC, gene ontology cellular component; GOMF, gene ontology molecular function
